# Supplementary material for: Anopheles coluzzii stearoyl-CoA desaturase is essential for adult female survival and reproduction upon blood feeding
Source: PLoS Pathog. 2021 May 20;17(5):e1009486. doi: 10.1371/journal.ppat.1009486 (PMC8171932; doi:10.1371/journal.ppat.1009486)
Supplement: S1 Fig — Alignment of the central domain of SCD1 (amino acid residues 88 to 303) and the equivalent domain of SCD1 orthologues in human, Drosophila melanogaster and Plasmodium falciparum, performed using CLUTALW. The three His boxes designated as region Ia, Ib and II, respectively, and the eight conserved His residues are shown. (DOCX) [file ppat.1009486.s001.docx]

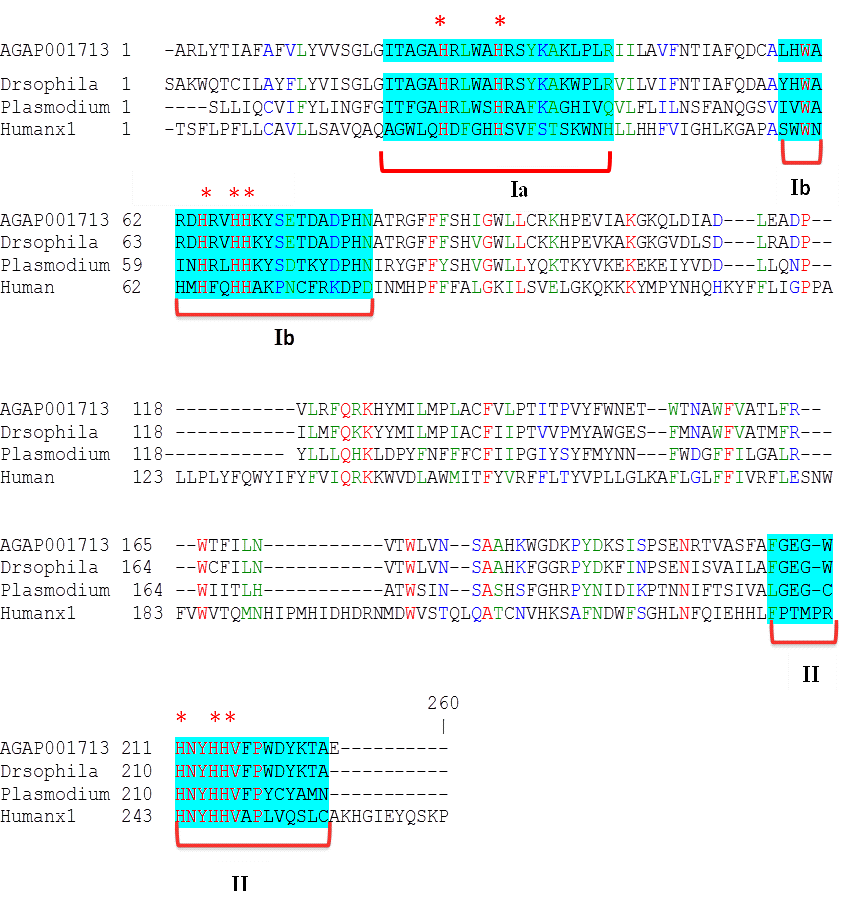


**S1 Fig. Sequence analysis of the *A. coluzzii* Stearoyl- CoA desaturase1.** Alignment of the central domain of SCD1 (amino acid residues 88 to 303) and the equivalent domain of SCD1 orthologues in human, *Drosophila melanogaster* and *Plasmodium falciparum*, performed using CLUTALW. The three His boxes designated as region Ia, Ib and II, respectively, and the eight conserved His residues are shown.
