# Supplementary material for: Anopheles coluzzii stearoyl-CoA desaturase is essential for adult female survival and reproduction upon blood feeding
Source: PLoS Pathog. 2021 May 20;17(5):e1009486. doi: 10.1371/journal.ppat.1009486 (PMC8171932; doi:10.1371/journal.ppat.1009486)
Supplement: S1 Table — (DOCX) [file ppat.1009486.s002.docx]

| **S1 Table. Putative stearoyl-CoA desaturases in the *A. gambiae* genome** | | | | | | |
| --- | --- | --- | --- | --- | --- | --- |
| **Domain: IPR015876**  **Acyl-CoA desaturase**  **Location: on 2R** | **Gene ID** | **Genome Location** | **Description (if known)** | **Target**  **%id** | **Query**  **%id** | **Genome**  **tBLASTn** |
|  | AGAP001713 | 2R: 8862946 | stearoyl-CoA desaturase (delta-9 desaturase)  [Source:VB Community Annotation] | NA | NA | 100% +  88% + 99% |
|  | AGAP013071 | 2R: 31439417 | Stearoyl-CoA desaturase 5  [Source:VB Community Annotation] | 39.90% | 44.51% | 54% |
| 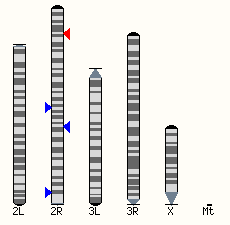 | AGAP003049 | 2R: 31443411 | stearoyl-CoA desaturase (delta-9 desaturase)  [Source:VB Community Annotation] | 38.56% | 43.66% | 54% + 54% |
|  | AGAP003050 | 2R: 31464582 | stearoyl-CoA desaturase (delta-9 desaturase)  [Source:VB Community Annotation] | 28.93% | 29.58% | No hit |
|  | AGAP003051 | 2R: 31471355 | stearoyl-CoA desaturase (delta-9 desaturase)  [Source:VB Community Annotation] | 41.99% | 45.07% | 50% |
|  | AGAP003418 | 2R: 37482847 | stearoyl-CoA desaturase (delta-9 desaturase)  [Source:VB Community Annotation] | 47.92% | 55.21% | 60% |
|  | AGAP004572 | 2R: 57680609 | No description | 30.59% | 47.04% | No hit |
|  | AGAP012720 | UNKN: 25105332 | No description | 32.08% | 14.37% | No hit |
