# Supplementary material for: Anopheles coluzzii stearoyl-CoA desaturase is essential for adult female survival and reproduction upon blood feeding
Source: PLoS Pathog. 2021 May 20;17(5):e1009486. doi: 10.1371/journal.ppat.1009486 (PMC8171932; doi:10.1371/journal.ppat.1009486)
Supplement: S2 Table — (DOCX) [file ppat.1009486.s003.docx]

| **S2 Table. Putative orthologues of *SCD1* in other mosquitoes and flies** | | | | |
| --- | --- | --- | --- | --- |
| **Species** | **Type** | **Orthologue** | **Target %id** | **Query %id** |
| *Anopheles arabiensis* | 1-to-1 | AARA002164 | 100.00% | 100.00% |
| *Anopheles coluzzii* | 1-to-1 | ACOM034558 | 100.00% | 100.00% |
| *Anopheles merus* | 1-to-1 | AMEM003516 | 100.00% | 100.00% |
| *Anopheles quadriannulatus* | 1-to-1 | AQUA000582 | 100.00% | 100.00% |
| *Anopheles melas* | 1-to-1 | AMEC009449 | 100.00% | 70.42% |
| *Anopheles epiroticus* | 1-to-1 | AEPI009927 | 98.87% | 98.87% |
| *Anopheles christyi* | 1-to-1 | ACHR006151 | 98.31% | 98.31% |
| *Anopheles funestus* | 1-to-1 | AFUN011754 | 98.31% | 98.31% |
| *Anopheles stephensi* | 1-to-1 | ASTE006887 | 98.03% | 98.03% |
| *Anopheles culicifacies* | 1-to-1 | ACUA024098 | 97.46% | 97.46% |
| *Anopheles minimus* | 1-to-1 | AMIN003522 | 97.18% | 97.18% |
| *Anopheles sinensis* | 1-to-1 | ASIS011562 | 96.90% | 96.90% |
| *Anopheles albimanus* | 1-to-1 | AALB003582 | 96.63% | 96.90% |
| *Anopheles darlingi* | 1-to-1 | ADAC010125 | 96.63% | 96.90% |
| *Anopheles atroparvus* | 1-to-1 | AATE007159 | 96.34% | 96.34% |
| *Anopheles dirus* | 1-to-1 | ADIR010381 | 96.34% | 96.34% |
| *Anopheles farauti* | 1-to-1 | AFAF019233 | 96.06% | 96.06% |
| *Culex quinquefasciatus* | 1-to-1 | CPIJ013748 | 89.58% | 89.58% |
| *Aedes aegypti* (LVP_AGWG) | 1-to-1 | AAEL003203 | 83.20% | 86.48% |
| *Musca domestica* | 1-to-many | MDOA013128 | 70.26% | 75.21% |
| *Drosophila melanogaster* | 1-to-many | Desat 1 (FBgn0086687) | 67.36% | 72.68% |
| *Musca domestica* | 1-to-many | MDOA000857 | 66.13% | 69.30% |
| *Drosophila melanogaster* | 1-to-many | Desat 2 (FBgn0043043) | 63.16% | 64.23% |
