# Supplementary material for: Anopheles coluzzii stearoyl-CoA desaturase is essential for adult female survival and reproduction upon blood feeding
Source: PLoS Pathog. 2021 May 20;17(5):e1009486. doi: 10.1371/journal.ppat.1009486 (PMC8171932; doi:10.1371/journal.ppat.1009486)
Supplement: S3 Table — (DOCX) [file ppat.1009486.s004.docx]

| **S3 Table. List of genes showing altered expression across timepoints in SCD1 KD mosquitoes** | | | | | | | |
| --- | --- | --- | --- | --- | --- | --- | --- |
| **Transcript ID** | **Functional Classification** | **Gene name** | **Fold changes** | | | | |
|  |  |  | **0h** | **6h** | **12h** | **18h** | **24h** |
| AGAP001942-RA | Carbohydrate and Lipid metabolism | Fumarylacetoacetase, C-terminal-related | -1.49 | -1.5 | -1.94965 | -2.1 | -2.07 |
| AGAP004352-RB | Carbohydrate and Lipid metabolism | succinyl-CoA synthetase beta subunit | -2.06 | -2.4 | -1.81 | -1.4 | -1.6 |
| AGAP001713-RB | Carbohydrate and Lipid metabolism | stearoyl-CoA desaturase (delta-9 desaturase) | -2.98 | -5.78 | -2.9 | -1.49 | -1.52 |
| AGAP009463-RA | Carbohydrate and Lipid metabolism | ATP-binding cassette transporter (ABC transporter) family G member | -1.31 | 1.057997 | -1.68 | -2.46 | -1.43 |
| AGAP008717-RA | Carbohydrate and Lipid metabolism | hydroxymethylglutaryl-CoA lyase | -1.57 | -2 | -1.33 | -1.298 | -1.43 |
| AGAP001713-RC | Carbohydrate and Lipid metabolism | stearoyl-CoA desaturase (delta-9 desaturase) | -3.22 | -4.5 | -2.73 | -1.3 | -1.4 |
| AGAP004352-RA | Carbohydrate and Lipid metabolism | succinyl-CoA synthetase beta subunit | -1.92 | -2.37 | -1.51 | -1.45 | -1.3 |
| AGAP006775-RA | Carbohydrate and Lipid metabolism | Glucosyl/glucuronosyl transferases | -1.18 | -1.21 | -2 | -1.45401 | -1.3 |
| AGAP006740-RA | Carbohydrate and Lipid metabolism | dolichyl-phosphate mannosyltransferase polypeptide 2, regulatory subunit | -2.01 | -3.96 | -2.12 | -1.24 | -1.26 |
| AGAP001200-RB | Carbohydrate and Lipid metabolism | glycogen debranching enzyme | -1.23 | -2.2 | -2.46 | -1.298 | -1.119 |
| AGAP001200-RA | Carbohydrate and Lipid metabolism | glycogen debranching enzyme | -1.11 | -1.5 | -2.24 | -1.1 | -1.03 |
| AGAP003049-RC | Carbohydrate and Lipid metabolism | stearoyl-CoA desaturase (delta-9 desaturase) | -1.36 | -2.35 | -1.52 | -1.27 | -1.03 |
| AGAP011984-RA | Carbohydrate and Lipid metabolism | UDP-N-acetyl-alpha-D-galactosamine:polypeptide N-acetylgalactosaminyltransferase 20 | -1.33 | 1.340404 | -1.11 | 2.079835 | 1.034114 |
| AGAP005504-RA | Carbohydrate and Lipid metabolism | phospholipid scramblase 1 | 1.069448 | 2.220635 | 2.10041 | 1.914016 | 1.472575 |
| AGAP005504-RD | Carbohydrate and Lipid metabolism | phospholipid scramblase 1 | 1.126298 | 2.035964 | 1.78991 | 1.873443 | 1.691984 |
| AGAP005504-RB | Carbohydrate and Lipid metabolism | phospholipid scramblase 1 | 1.210008 | 2.226592 | 1.755443 | 2.110448 | 1.694627 |
| AGAP010695-RA | Carbohydrate and Lipid metabolism | elongation of very long chain fatty acids protein 4 | 1.206646 | -1.1 | -1.15 | -1.4 | 2.084245 |
| AGAP011948-RA | Carbohydrate and Lipid metabolism | threonine 3-dehydrogenase | -1.05 | -1.409 | 1.178857 | 1.571312 | 2.291269 |
| AGAP002032-RA | Carbohydrate and Lipid metabolism | Lipid transport protein | 1.507056 | 1.350371 | 1.431939 | 2.067538 | 2.704781 |
| AGAP004426-RB | Cytoskeleton / Cell adhesion / Structural components | FAS1 domain | 1.087899 | 1.050508 | -1.278 | -1.43834 | -2.03 |
| AGAP005360-RA | Cytoskeleton / Cell adhesion / Structural components | PQ loop repeat-containing protein 3 | -1.054 | 1.009671 | -2.3 | -1.26 | 1.191178 |
| AGAP005095-RA | Cytoskeleton / Cell adhesion / Structural components | actin beta/gamma 1 | -1.08 | 2.042297 | 1.067581 | -1.27 | 1.296873 |
| AGAP004935-RA | Cytoskeleton / Cell adhesion / Structural components | Ankyrin repeat | 1.050927 | 2.277828 | 1.375176 | 1.620239 | 1.355118 |
| AGAP006148-RA | Cytoskeleton / Cell adhesion / Structural components | CPLCA3 | 1.018452 | -1.17 | 1.904501 | 2.109506 | 1.376727 |
| AGAP009200-RB | Cytoskeleton / Cell adhesion / Structural components | collagen, type IV, alpha | 1.823213 | 1.4458 | 1.501694 | 1.579754 | 2.103669 |
| AGAP000969-RB | Cytoskeleton / Cell adhesion / Structural components | tropomodulin | 1.212274 | 1.396145 | -1.3 | 1.394434 | 2.262444 |
| AGAP007103-RD | Cytoskeleton / Cell adhesion / Structural components | Calsyntenin-1 | -1 | 1.233959 | 1.334675 | 1.479464 | 2.285695 |
| AGAP001381-RA | Cytoskeleton / Cell adhesion / Structural components | laminin, beta 1 | 1.398535 | 1.655494 | 1.097785 | 1.266421 | 2.349416 |
| AGAP009790-RA | Cytoskeleton / Cell adhesion / Structural components | CPAP3 | 1.511569 | 1.113605 | 2.099975 | 1.434819 | 3.256924 |
| AGAP006647-RB | Immunity / Putative immunity / Phagocytosis | Leucine-rich repeat domain | -1.22 | 1.063556 | -1.077 | -1.067 | -2.08 |
| AGAP006647-RA | Immunity / Putative immunity / Phagocytosis | Leucine-rich repeat domain | -1.32 | -1.04 | -1.11 | -1.14 | -2.07 |
| AGAP008654-RA | Immunity / Putative immunity / Phagocytosis | TEP12 | -1.3 | -1.181 | -2 | -1.256 | -1.2 |
| AGAP001769-RB | Immunity / Putative immunity / Phagocytosis | Beat protein | 1.688098 | 1.483634 | 1.836994 | 2.156669 | 1.132659 |
| AGAP006909-RA | Immunity / Putative immunity / Phagocytosis | SPRN1 | 1.473078 | 2.235072 | 1.560846 | 1.372 | 1.160503 |
| AGAP001769-RD | Immunity / Putative immunity / Phagocytosis | Beat protein | 1.978516 | 2.083454 | 1.952135 | 2.104706 | 1.163552 |
| AGAP001769-RC | Immunity / Putative immunity / Phagocytosis | Beat protein | 1.764019 | 2.100596 | 1.508109 | 2.080967 | 1.311774 |
| AGAP000720-RB | Cytoskeleton / Cell adhesion / Structural components | Neuronal cell adhesion molecule | 2.678655 | 1.102464 | 1.210577 | -1.03 | 1.360428 |
| AGAP005246-RE | Immunity / Putative immunity / Phagocytosis | SRPN10 | -1.06 | 2.150949 | -1.1 | 1.348671 | 1.439932 |
| AGAP009215-RA | Immunity / Putative immunity / Phagocytosis | CLIPB18 | 1.526035 | 1.814823 | 1.287792 | 1.776654 | 2.179525 |
| AGAP003610-RA | Immunity / Putative immunity / Phagocytosis | Immunoglobulin I-set | 1.902506 | 1.696711 | 2.173595 | 1.712431 | 2.260365 |
| AGAP007209-RB | Immunity / Putative immunity / Phagocytosis | Tetraspanin | 1.689261 | 2.275483 | 1.271376 | 1.701983 | 2.336936 |
| AGAP002422-RA | Immunity / Putative immunity / Phagocytosis | CLIPD1 | 1.398917 | 1.37124 | 1.585267 | 1.802481 | 2.566489 |
| AGAP008927-RA | Immunity / Putative immunity / Phagocytosis | protein TILB homolog | -1 | 1.020153 | 1.340146 | 3.754717 | 3.16982 |
| AGAP012757-RA | Protein degradation / Proteasome | Aminopeptidase N1 | -2.89 | -1 | -1.47 | -1.788 | -3.97 |
| AGAP000901-RA | Protein degradation / Proteasome | Alanine transaminase | -2.2 | -1.44 | -1.54 | -1.987 | -2.35 |
| AGAP002720-RA | Protein degradation / Proteasome | Cathepsin O | -2.05 | -1.347 | -1.38 | -1.4 | -1 |
| AGAP012662-RA | Protein degradation / Proteasome | Omega-amidase | -1.6 | -1.63 | -2.048 | -1.11 | -1.052 |
| AGAP009266-RA | Protein degradation / Proteasome | Low molecular weight phosphotyrosine protein phosphatase | -1.07 | 1.256108 | -1.14 | 1.13554 | 2.086455 |
| AGAP002878-RA | Protein degradation / Proteasome | Cystatin-like protein | 1.491348 | 2.055883 | 1.006411 | 1.56786 | 2.23069 |
| AGAP011909-RA | Protein degradation / Proteasome | Peptidase S1 | 1.199041 | 1.164367 | 1.785166 | 1.593683 | 2.449878 |
| AGAP010885-RA | Redox / Apoptosis / Detoxification | (S)-2-hydroxy-acid oxidase | -1.67032 | -1.07963 | -1.80772 | -2.1667 | -2.41845 |
| AGAP003785-RD | Redox / Apoptosis / Detoxification | glucose dehydrogenase (acceptor) | -1.48791 | -1.03926 | -1.33031 | -1.34718 | -2.40031 |
| AGAP006023-RA | Redox / Apoptosis / Detoxification | tyrosine 3-monooxygenase | -1.00436 | -1.10172 | 1.001112 | -1.3767 | -2.025 |
| AGAP005009-RA | Redox / Apoptosis / Detoxification | pyrroline-5-carboxylate reductase | -2.15985 | -1.33513 | -1.6371 | -1.11284 | -1.37597 |
| AGAP000109-RA | Redox / Apoptosis / Detoxification | cytochrome c oxidase subunit VIIa | -1.24869 | -2.38858 | -1.51732 | -1.27944 | -1.359 |
| AGAP003167-RA | Redox / Apoptosis / Detoxification | NAD(P) transhydrogenase | -2.21316 | -1.93682 | -1.79971 | -1.76453 | -1.22634 |
| AGAP011507-RA | Redox / Apoptosis / Detoxification | COE13O | -2.70626 | -2.35412 | -2.71888 | -1.37181 | 1.031038 |
| AGAP009783-RA | Redox / Apoptosis / Detoxification | short/branched chain acyl-CoA dehydrogenase | -1.20807 | -2.05442 | -1.5835 | -1.11933 | 1.202935 |
| AGAP011334-RA | Redox / Apoptosis / Detoxification | Failed axon connections protein | 1.265732 | 2.36361 | 1.599421 | 1.444883 | 1.485407 |
| AGAP000327-RA | Redox / Apoptosis / Detoxification | tyrosine aminotransferase | 1.167939 | 1.457156 | 1.082345 | 1.592865 | 2.089807 |
| AGAP011066-RA | Redox / Apoptosis / Detoxification | Aldose reductase | 1.001711 | 1.085757 | 1.076431 | 1.182333 | 2.205956 |
| AGAP004163-RB | Redox / Apoptosis / Detoxification | GSTD7 | 1.57039 | 1.707788 | 1.700157 | 3.093798 | 2.491309 |
| AGAP004592-RI | Replication/ Transcription/ Translation / Transcription factors / Cell cycle | splicing factor, arginine/serine-rich 4/5/6 | -1.46409 | -1.24769 | -1.15852 | -2.10307 | -2.59803 |
| AGAP004592-RA | Replication/ Transcription/ Translation / Transcription factors / Cell cycle | splicing factor, arginine/serine-rich 4/5/6 | -1.49885 | -1.44273 | -1.23606 | -1.94727 | -2.59297 |
| AGAP005336-RA | Replication/ Transcription/ Translation / Transcription factors / Cell cycle | SWI/SNF-related matrix-associated actin-dependent regulator of chromatin subfamily D member 1 | -1.07713 | -1.12366 | -1.04774 | -1.44314 | -2.36242 |
| AGAP005127-RA | Replication/ Transcription/ Translation / Transcription factors / Cell cycle | RNA-binding protein 15 | -1.14069 | -1.15556 | -1.00814 | -1.3185 | -2.22773 |
| AGAP004592-RH | Replication/ Transcription/ Translation / Transcription factors / Cell cycle | splicing factor, arginine/serine-rich 4/5/6 | -1.58048 | -1.09617 | -1.03162 | -1.74247 | -2.22488 |
| AGAP006702-RB | Replication/ Transcription/ Translation / Transcription factors / Cell cycle | No metadata available | -1.01439 | 1.037068 | -1.48988 | -1.06938 | -2.06748 |
| AGAP012413-RA | Replication/ Transcription/ Translation / Transcription factors / Cell cycle | CycA | -1.11569 | 1.099563 | 1.341263 | -1.40038 | -2.04775 |
|  |  |  |  |  |  |  |  |
| AGAP007967-RA | Replication/ Transcription/ Translation / Transcription factors / Cell cycle | selenide, water dikinase | -1.19435 | -2.58662 | 1.050573 | -1.68258 | -1.87734 |
| AGAP005122-RA | Replication/ Transcription/ Translation / Transcription factors / Cell cycle | UBX domain-containing protein 1 | 1.009402 | 1.003768 | -1.3122 | -2.32226 | -1.5898 |
| AGAP003912-RA | Replication/ Transcription/ Translation / Transcription factors / Cell cycle | histone H2B | 1.022605 | -1.37349 | 2.035367 | 1.02038 | -1.29027 |
| AGAP000179-RC | Replication/ Transcription/ Translation / Transcription factors / Cell cycle | Amidophosphoribosyltransferase | -1.45003 | -3.98579 | -2.08107 | -1.19225 | -1.06942 |
| AGAP000179-RB | Replication/ Transcription/ Translation / Transcription factors / Cell cycle | Amidophosphoribosyltransferase | -1.41135 | -2.78225 | -1.65886 | -1.09555 | 1.06507 |
| AGAP000179-RA | Replication/ Transcription/ Translation / Transcription factors / Cell cycle | Amidophosphoribosyltransferase | -1.42266 | -3.9325 | -1.66248 | -1.13039 | 1.092706 |
| AGAP001544-RA | Replication/ Transcription/ Translation / Transcription factors / Cell cycle | tripartite motif-containing protein 37 | 1.423844 | 1.020083 | 2.028657 | 1.588341 | 1.177675 |
| AGAP003913-RA | Replication/ Transcription/ Translation / Transcription factors / Cell cycle | histone H2A | -1.06758 | -1.02007 | -1.10408 | 1.153308 | 2.0182 |
| AGAP007103-RB | Replication/ Transcription/ Translation / Transcription factors / Cell cycle | Calsyntenin-1 | -1.06788 | 1.20135 | 1.2666 | 1.331525 | 2.035862 |
| AGAP003911-RA | Replication/ Transcription/ Translation / Transcription factors / Cell cycle | histone H2A | 1.093662 | 1.019489 | -1.19246 | -1.05034 | 2.094304 |
| AGAP002670-RB | Replication/ Transcription/ Translation / Transcription factors / Cell cycle | Zinc finger, ZZ-type | 1.635649 | 1.897166 | 1.74999 | 1.492876 | 2.187532 |
| AGAP010600-RA | Signaling / ATPases / GTPases | Insulin-like peptide 2 precursor | -1.23174 | 1.107282 | -1.17156 | -1.29054 | -2.1499 |
| AGAP003720-RA | Signaling / ATPases / GTPases | Annexin A4 | 1.008065 | 2.211569 | 1.664695 | -1.05379 | -1.33895 |
| AGAP005627-RC | Signaling / ATPases / GTPases | creatine kinase | -1.06868 | 2.025038 | 1.242582 | -1.23439 | 1.165271 |
| AGAP000297-RA | Signaling / ATPases / GTPases | No metadata available | 1.457739 | 2.408736 | 1.899182 | 1.88105 | 1.475471 |
| AGAP000617-RB | Signaling / ATPases / GTPases | dual specificity phosphatase | 1.182302 | 1.188406 | 1.678801 | 1.84419 | 2.013438 |
| AGAP002905-RA | Signaling / ATPases / GTPases | OBP13 | 1.215234 | -1.02322 | 1.084529 | 1.433635 | 2.041939 |
| AGAP013136-RA | Signaling / ATPases / GTPases | ATPase | 1.317061 | 1.607666 | 1.442288 | 1.281049 | 2.048399 |
| AGAP002858-RC | Signaling / ATPases / GTPases | sodium/potassium-transporting ATPase subunit alpha | 1.430299 | 2.146422 | 1.603715 | 1.351142 | 2.057086 |
| AGAP005162-RB | Signaling / ATPases / GTPases | dystroglycan 1 | 2.260792 | 1.642941 | 1.928089 | 1.691522 | 2.061215 |
| AGAP010225-RA | Signaling / ATPases / GTPases | ER lumen protein retaining receptor | 1.475634 | 1.743161 | -1.07569 | 1.287575 | 2.114949 |
| AGAP003121-RA | Signaling / ATPases / GTPases | phosphatidylinositol 4-kinase type 2 | 1.701343 | 1.701883 | 1.842416 | 1.506845 | 2.233235 |
| AGAP012755-RA | Signaling / ATPases / GTPases | ER lumen protein retaining receptor | 1.450365 | 1.625054 | -1.09839 | 1.475648 | 2.240455 |
| AGAP012321-RA | Signaling / ATPases / GTPases | OBP26 | -1.05546 | 1.131918 | 1.20807 | 1.1504 | 2.269414 |
| AGAP001573-RA | Signaling / ATPases / GTPases | Ras-related C3 botulinum toxin substrate 1 | 1.567719 | 1.881858 | 1.714255 | 1.64289 | 2.286472 |
| AGAP008054-RA | Signaling / ATPases / GTPases | chemosensory protein | -1.04478 | -2.37925 | 1.433397 | 2.273071 | 2.410843 |
| AGAP012302-RA | Transport / Vesicule mediated transport | Sodium-independent sulfate anion transporter | -1.57693 | -1.05192 | -2.29933 | -1.04725 | -1.63552 |
| AGAP005563-RC | Transport / Vesicule mediated transport | Tret1 | -2.10731 | -1.09866 | -1.68863 | 1.059881 | -1.15982 |
| AGAP010975-RA | Transport / Vesicule mediated transport | Sodium/potassium/calcium exchanger | -1.28861 | -1.10975 | -2.25794 | -1.37641 | -1.01364 |
| AGAP004519-RB | Transport / Vesicule mediated transport | Sideroflexin 1,2,3 | -1.43511 | -2.57205 | -1.32659 | 1.121886 | 1.078727 |
| AGAP001487-RA | Transport / Vesicule mediated transport | innexin shaking-B | 1.190948 | 1.138892 | 1.267057 | 2.038185 | 1.940026 |
| AGAP011062-RA | Transport / Vesicule mediated transport | Hemoglobin (Heterodimeric) | 1.595408 | 2.319121 | 1.482087 | 1.454539 | 1.956269 |
| AGAP005933-RA | Transport / Vesicule mediated transport | NFkappaB essential modulator | 1.287767 | 2.175518 | 1.765074 | 1.439133 | 2.016453 |
| AGAP001550-RA | Transport / Vesicule mediated transport | Sodium-coupled monocarboxylate transporter 1 | 1.108476 | 1.059807 | -1.09976 | 1.260383 | 2.030383 |
| AGAP004896-RA | Transport / Vesicule mediated transport | potassium channel, subfamily K, member 2 | 1.356011 | 1.085395 | 1.024606 | 1.319575 | 2.044282 |
| AGAP000128-RA | Transport / Vesicule mediated transport | MFS transporter, VNT family, synaptic vesicle glycoprotein 2 | -1.14218 | 1.114404 | 1.074194 | 1.365641 | 2.393502 |
| AGAP000242-RA | Transport / Vesicule mediated transport | vesicle transport protein SEC20 | 2.537641 | 1.647824 | 1.919043 | 1.498809 | 2.703308 |
| AGAP006123-RA | Unknown | No metadata available | -1.30829 | -1.22385 | -2.03879 | -2.14118 | -3.53638 |
| AGAP000867-RA | Unknown | No metadata available | -1.09293 | 1.079907 | -1.13828 | -1.32676 | -2.11095 |
| AGAP012609-RA | Unknown | No metadata available | -1.20116 | -1.4271 | -1.38345 | -1.99635 | -2.05029 |
| AGAP005585-RC | Unknown | No metadata available | -1.4192 | -1.26974 | -2.1081 | -1.51304 | -1.52844 |
| AGAP001890-RA | Unknown | No metadata available | -2.1215 | -1.03305 | -1.54515 | -1.36342 | -1.39432 |
| AGAP003550-RA | Unknown | No metadata available | 1.032961 | -1.12179 | 2.077075 | -1.15416 | -1.33663 |
| AGAP000964-RA | Unknown | No metadata available | 1.383569 | 2.047982 | 1.238912 | 1.480238 | 1.286986 |
| AGAP005379-RA | Unknown | No metadata available | 2.19132 | 1.606998 | 1.631871 | 1.311797 | 1.355775 |
| AGAP001718-RA | Unknown | No metadata available | 1.205768 | 2.114587 | 1.362836 | 1.416854 | 1.65515 |
| AGAP003352-RC | Unknown | No metadata available | 1.238621 | 2.082348 | 1.523097 | 1.452471 | 1.699941 |
| AGAP013481-RA | Unknown | No metadata available | -1.25197 | 2.07281 | -1.30042 | 1.168635 | 1.807216 |
| AGAP010045-RA | Unknown | No metadata available | 1.773176 | 1.425342 | 2.022611 | 1.692747 | 1.89711 |
| AGAP006971-RA | Unknown | No metadata available | 2.008594 | 1.971022 | 1.573981 | 1.551044 | 1.959979 |
| AGAP007185-RA | Unknown | No metadata available | 1.668549 | 1.803589 | 1.522357 | 1.434002 | 2.01494 |
| AGAP005813-RA | Unknown | No metadata available | 1.145619 | 1.476206 | 1.3618 | 1.462489 | 2.032202 |
| AGAP010148-RA | Unknown | No metadata available | 1.948678 | 1.593487 | 1.39269 | 1.829059 | 2.133985 |
| AGAP011044-RA | Unknown | No metadata available | 1.122885 | 1.656325 | -1.04336 | 1.647084 | 2.138512 |
| AGAP002085-RC | Unknown | No metadata available | -1.01175 | 1.75586 | 1.30576 | 1.836324 | 2.179714 |
| AGAP000697-RA | Unknown | No metadata available | 1.953204 | 1.316351 | 1.715757 | 1.301095 | 2.189728 |
| AGAP002085-RA | Unknown | No metadata available | -1.08349 | 1.70638 | 1.284027 | 1.387705 | 2.209175 |
| AGAP002085-RB | Unknown | No metadata available | 1.078314 | 1.415684 | 1.368592 | 1.617977 | 2.308576 |
| AGAP013076-RA | Unknown | No metadata available | 1.380929 | 1.662653 | 1.344467 | 1.197377 | 2.312475 |
| AGAP010066-RA | Unknown | No metadata available | 1.300035 | 2.238816 | -1.13792 | 1.675771 | 2.567861 |
| AGAP001174-RA | Unknown | No metadata available | 2.433242 | 3.20621 | 2.72398 | 2.438771 | 2.640051 |
| AGAP007842-RA | Unknown | No metadata available | 1.868415 | 2.373607 | 2.60963 | 1.700773 | 2.664279 |
| AGAP003060-RA | Unknown | No metadata available | 2.495242 | 1.489915 | 2.534426 | 1.728571 | 2.785277 |
| AGAP009462-RA | Unknown | No metadata available | 2.199095 | 1.92223 | 1.781883 | 1.7207 | 3.328377 |
| AGAP011576-RA | Unknown | No metadata available | 2.691398 | 2.044409 | 2.013618 | 1.836891 | 3.430441 |
| AGAP003773-RA | Unknown | No metadata available | 1.333928 | 1.87219 | -1.12195 | 2.026548 | 3.841509 |
| AGAP007314-RA | Unknown | No metadata available | 2.873416 | 1.75405 | 1.504276 | 3.147106 | 5.37099 |
| AGAP011065-RA | Unknown | No metadata available | 1.043243 | -1.34945 | 1.603437 | 2.371093 | 5.633541 |
