# Supplementary material for: Anopheles coluzzii stearoyl-CoA desaturase is essential for adult female survival and reproduction upon blood feeding
Source: PLoS Pathog. 2021 May 20;17(5):e1009486. doi: 10.1371/journal.ppat.1009486 (PMC8171932; doi:10.1371/journal.ppat.1009486)
Supplement: S4 Table — (DOCX) [file ppat.1009486.s005.docx]

| **S4 Table. List of immune genes showing differential expression in SCD1 KD mosquitoes** | | | | | | |
| --- | --- | --- | --- | --- | --- | --- |
| **Gene ID** | **Gene name** | **Fold change** | | | | |
|  |  | **0h** | **6h** | **12h** | **18h** | **24h** |
| AGAP011787-RA | CLIPA5 | 4.637564 | 4.176203 | No data | 1.692576 | 3.608658 |
| AGAP010731-RA | CLIPA8 | 1.060295 | No data | 1.328005 | No data | 2.295051 |
| AGAP009214-RA | CLIPB11 | 1.415461 | No data | No data | 1.907089 | 2.248525 |
| AGAP009217-RA | CLIPB12 | 4.878154 | No data | 2.448646 | No data | 2.879107 |
| AGAP010833-RA | CLIPB14 | 3.052399 | No data | 1.656077 | 1.800905 | 2.373628 |
| AGAP009844-RA | CLIPB15 | 1.834629 | No data | No data | 2.1827 | 3.402466 |
| AGAP001648-RA | CLIPB17 | 5.261095 | No data | 4.61839 | 4.093173 | No data |
| AGAP009215-RA | CLIPB18 | 1.526035 | 1.814823 | 1.287792 | 1.776655 | 2.179525 |
| AGAP003247-RA | CLIPB19 | 1.018067 | 1.244041 | No data | 2.832054 | 1.736463 |
| AGAP012037-RA | CLIPB20 | 1.223776 | 2.076342 | No data | -1.09205 | 1.019822 |
| AGAP002422-RA | CLIPD1 | 1.398917 | 1.37124 | 1.585267 | 1.802481 | 2.566488 |
| AGAP007456-RA | LRRIM8B | No data | -2.49836 | -2.36044 | No data | No data |
| AGAP007455-RA | LRRIM10 | -1.35984 | No data | -2.78161 | No data | No data |
| AGAP010816-RA | TEP3 | 2.456022 | No data | 1.9697 | 2.094799 | 4.355418 |
| AGAP008654-RA | TEP12 | 1.311118 | 1.181009 | 2.007956 | 1.256317 | 1.203668 |
| AGAP010811-RA | FREP19 | No data | 1.132828 | 1.739411 | 5.656849 | No data |
| AGAP010763-RA | FREP21 | No data | 1.655453 | 1.065637 | 2.88555 | 3.027795 |
| AGAP011228-RA | FREP24 | No data | No data | 4.051735 | No data | No data |
| AGAP010759-RA | FREP32 | No data | 2.443725 | 1.668615 | No data | 2.673858 |
| AGAP011231-RA | FREP59 | No data | 2.08607 | 1.018616 | 1.164993 | 1.435175 |
| AGAP012352-RA | ML1 | -1.85754 | No data | -4.97349 | No data | No data |
| AGAP002804-RA | ML4 | No data | No data | No data | 3.715127 | No data |
| AGAP002849-RA | ML7 | No data | No data | 1.315672 | 9.261548 | 6.937465 |
| AGAP004017-RA | Leucine rich protein | 1.604855 | No data | 1.777525 | 2.573785 | 2.989334 |
| AGAP006647-RA | Leucine rich protein | 1.323321 | 1.046415 | 1.112405 | 1.149128 | 2.027941 |
| AGAP000693-RA | CEC-1 | 3.640545 | No data | 2.455529 | 2.327022 | 4.479035 |
| AGAP005334-RA | CTLMA2 | 2.149406 | No data | 1.483791 | 2.090924 | 2.619961 |
| AGAP004247-RC | GPXH1 | 1.25162 | 2.064726 | No data | 1.618945 | 1.587142 |
| AGAP005848-RA | Ficolin-A | 1.106999 | 3.143801 | 1.788332 | No data | 1.365175 |
| AGAP008179-RA | SCRBQ3 | No data | No data | No data | 2.314181 | No data |
| AGAP004975-RA | PPO3 | No data | No data | 1.191027 | 1.026363 | 2.979984 |
| AGAP009200-RB | Collagen IV | 1.823213 | 1.4458 | 1.501695 | 1.579754 | 2.10367 |
| AGAP007159-RA | Alpha-crystallin B chain | No data | No data | 1.009312 | 1.3502 | 2.077811 |
| AGAP008645-RA | GAM1 | No data | 1.291917 | No data | 1.762335 | 2.112557 |
| AGAP001212-RA | PGRPLB1 | No data | No data | No data | No data | 2.3273 |
| AGAP001212-RB | PGRLB2 | No data | No data | No data | No data | 2.237026 |
| AGAP007209-RB | TETRASPANIN | 1.689261 | 2.275483 | 1.271377 | 1.701983 | 2.336935 |
| AGAP010759-RA | TETRASPANIN | No data | 2.443725 | 1.668615 | No data | 2.673858 |
